# A Scoping Review of Empirical Research on Research Ethics Board Membership and Expertise

**DOI:** 10.1177/15562646251376747

**Published:** 2025-10-01

**Authors:** Emma Tumilty, Jake Young, Richard James, Kimberley Serpico, Ann Johnson, Emily E. Anderson

**Affiliations:** 1 Department of Bioethics and Health Humanities, School of Population and Public Health, 12338 University of Texas Medical Branch, Galveston, Texas, USA; 2 2445 American Medical Association, Chicago, IL, USA; 3 Nemours Medical Libraries, Nemours Children's Health, Wilmington, Delaware, USA; 4 Office of Regulatory Affairs and Research Compliance, 1857 Harvard T. H. Chan School of Public Health, Boston, Massachusetts, USA; 5 Institutional Review Board & Human Research Protections Program, University of Utah, East Salt Lake City, Utah, USA; 6 Neiswanger Institute for Bioethics, Stritch School of Medicine, 2456 Loyola University Chicago, Maywood, IL, USA

**Keywords:** research ethics committee/IRB review, IRB performance/Quality/Assessment/Evaluation, research ethics, other, federal policies/Guidelines/Office of human research protections

## Abstract

REB membership and its local idioculture play a key role in the decisions made. Little evidence exists as to what composition of membership expertise and training creates the conditions for a board to be most effective. This scoping review of the empirical research on REB membership and expertise aims to outline what evidence has been gathered and what gaps exist. Our main research question was: What empirical research exists on how research ethics boards (REBs) identify and train members and ensure they have adequate expertise to review research protocols? We found a small and diverse body of literature from around the world. We summarized findings in four themes: scientific expertise, ethical, legal and regulatory training and expertise, diversity of identity and perspectives, and engagement with research participant perspectives. Studies reviewed identified issues for all aspects of membership expertise and training. Further work is needed to establish best practices.

## Introduction

Research ethics boards (REBs) are tasked with the protection of the rights and welfare of human subjects (CIOMS, 2016). They fulfill this task through a review of protocols and then make a decision regarding approval—with or without minor modifications—or rejection. They may also require subsequent monitoring of research activities depending on the nature of the study. Since their inception in the mid- to late twentieth century, REBs have constituted a form of peer review “plus” undertaken by largely scientific and clinical experts, as well as lay members or community representatives (Hedgecoe, 2020). REBs exist across the world with varied nomenclature, operational aspects, and membership (OHRP-International, 2022).

Most countries have specific guidance regarding REB membership and its diversity and expertise requirements in their research regulations. In terms of the international guidance, CIOMS Guideline 23: Requirements for Establishing Research Ethics Committees and their Review of Protocols, states that REBs:

(…) must include multidisciplinary membership in order to competently review the proposed research. Committee members must be duly qualified and regularly update their knowledge of ethical aspects of health-related research (…). Membership normally must include physicians, scientists and other professionals such as research coordinators, nurses, lawyers, and ethicists, as well as community members or representatives of patients’ groups who can represent the cultural and moral values of study participants. Ideally, one or more members should have experience as study participants since there is growing recognition that knowledge gained through personal experience as a participant can supplement the professional understanding of illness and medical care. Committees must include both men and women. When a proposed study involves vulnerable individuals or groups, as may be the case in research involving prisoners or illiterate persons, representatives of relevant advocacy groups should be invited to meetings where such protocols will be reviewed. (CIOMS, 2016)

Although CIOMS guidelines may be considered aspirational, the recommendations that REBs should be diverse in terms of demographics; disciplinary/content expertise spanning science, ethics, and law; and inclusive of multiple stakeholder perspectives are clearly aligned with the responsibilities of REBs to protect participants and promote high-quality, ethical research. The CIOMS guidelines are also echoed in many national-level regulations such as in the United States in the Common Rule (45 CFR §46.107), in Australia in the National Statement (Section 5.1.30, 2023) (National Health and Medical Research Council, Australian Research Council and Universities Australia, 2023), or in Malaysia's Guideline for Independent Ethics Committee Registration and Inspection (NPCB, 2016).

REB members’ identity and expertise influences their decision-making, not only in relation to whether or not to approve a project, but also in relation to changes they may request regarding language in consent documents, safety monitoring, or means of addressing concerns such as undue inducement (Stark, 2012). The composition of REB membership and its local idioculture therefore play a key role in the decisions it makes (Pritchard, 2011; Stark, 2012; Tolich, 2015). Little evidence exists as to what membership or training creates the conditions for a board to be most effective. Previous research suggests that REBs privilege scientific expertise over other kinds of expertise (MacNeill et al., 1994). Despite this, however, concerns have long been raised that REBs do not have adequate scientific expertise (Klitzman, 2015; Milford et al., 2006; Paul, 2000; Schrag, 2010; Stark, 2012). In addition, there is a growing recognition of the importance of adequately representing the perspectives of research participants and their communities in the research review process (Anderson et al., 2006; Hedgecoe, 2020). However, the way that this is best achieved or can be assessed is less clear.

This scoping review of the empirical research on REB membership and expertise aims to outline what research questions have been asked, what evidence has been gathered, and what gaps exist in the research literature. Our main research question was: What empirical research exists on how research ethics boards (REBs) identify and train members and ensure they have adequate expertise to review research protocols? Given the multifaced nature of REB expertise and the requirements of international regulations, we refined our inquiry to focus on four specific questions:

1. How do REBs ensure they have appropriate scientific expertise?
  Each REB requires sufficient scientific expertise to understand whether a protocol under review is responsibly designed to yield useful scientific and/or social information. While there is debate about whether an REB should engage in scientific review (Hedgecoe, 2020; Klitzman, 2015), a degree of scientific validity has to be established by some means for a project to reach a minimum threshold of justification in a risk-versus -benefit equation. To achieve this, REBs require their members to have sufficient knowledge of the methods used in the institution's core research portfolio to make these judgements during review.
2. How do REBs ensure they have appropriate ethical, legal, and regulatory expertise?
  In most countries, REB members undertake some limited form of training after becoming an REB member. This may be a workshop, online modules, or more extensive training and may be focused on regulation and/or ethical analysis. In addition, REBs may have a (bio)ethics expert as one of their members, though this is generally not a regulatory requirement and depends on local access. Legal expertise is similarly varied in its provision and can depend on local regulation and access. Regulatory expertise, while part of the REBs remit, is often provided by administrative/support staff or HRPP program staff (Babb, 2020).
3. How do REBs ensure they have appropriate diversity of people and perspectives?
  Diversity in REB membership can mean both diversity in identity (e.g., race, ethnicity, class, gender, sexual orientation, disability, etc.) of REB members, but also diversity in member types such as lay, non-scientist, or community members as well as different kinds of scientist members. Many regulations that govern REBs specify some mix of member identity (although this is not universal) and regulations in many countries require that there be at least one community, lay, or non-scientific member (or various combinations of these) (Emanuel et al., 2008; Hernandez et al., 2009).
4. How do REBs ensure that research participants’ perspectives are appropriately represented? There are no formal requirements to include previous research participants in the membership of REBs. Many regulations do require the inclusion of lay/non-scientist or community members, who are thought to, in some way, represent potential research participant views and experiences.

## Methods

Empirical research on REBs is sparse and disparate (Grady, 2019). Scoping reviews are useful to describe the current state of research literature on a given topic and identify research gaps (Arksey & O’Malley 2005; Levac et al., 2010). Scoping reviews are also an effective strategy when bodies of literature cross disciplines and methods, as is the case for research and scholarship on REB practices. In the last decade, there have been several scoping reviews published related to REB practice (Nicholls et al. 2015) and community engagement and research ethics (Mikesell et al., 2013; Zhao et al., 2019), but none has focused specifically on REB membership and expertise in an international context.

We employed a five-step framework for a scoping review modified from Levac et al. (2010): 1) identify the research questions; 2) identify relevant studies; 3) select relevant studies; 4) chart the data from relevant studies; 5) and collate, summarize, and report the results.

### Identifying Relevant Studies

Studies were identified through an electronic search of published literature, supplemented by hand-searching of seven key research ethics/bioethics journals starting with earliest date of publication (*Research Ethics, American Journal of Bioethics (AJOB), AJOB Empirical Research, Accountability in Research, Science and Engineering Ethics, Ethics & Human Research* (formerly *IRB: Ethics & Human Research*) and the *Journal of Empirical Research on Human Research Ethics (JERHRE))* and tracking of citations in identified articles. A Masters-trained research librarian (RJ) conducted electronic searches of the databases PubMed, PsychINFO, Scopus, and Google Scholar. Terms relating to “research ethics boards” were combined with terms relating to “board composition and expertise” (see Appendix A for full list of search terms). The search strategy was developed by the authors, who are experts in research ethics and REB policy, management, and function, in consultation with the research librarian. The search strategy included both Medical Subject Heading (MeSH) terms (PubMed) and keywords.

We developed specific eligibility criteria for articles based on our research questions. As our primary motivation for conducting the scoping review was to advance the research agenda on REB expertise, we limited our search to include only articles that reported results of empirical research focused on expertise and identification, selection or training of REB members, and staff, or consultants to assist in review. We excluded articles that did not involve empirical research or did not have REB membership or expertise as a core element of their study. Articles were not limited by date or country; only English language studies were included.

### Study Selection

The electronic search and hand-searches were conducted in February 2022. All articles were imported into Zotero for tracking and organization. All titles and abstracts were initially screened by at least two reviewers; articles were rejected if reviewers could determine that the articles did not meet the inclusion criteria. Three authors reviewed the full texts to make the final determination regarding inclusion.

### Charting and Summarizing the Data

Guided by the four research questions, the senior author (Anderson) drafted the data charting form which was then revised with input from the rest of the team. Through this process, we determined which variables to extract to best answer our research questions and to describe the research landscape related to REB expertise. Once the data charting form was finalized, all articles were reviewed and charted independently by two team members. First, we created descriptive summaries of data from charting forms that included numerical analysis and qualitative thematic analysis. The second step was to describe results in response to each specific research question. Lastly, we considered implications of the findings for REB practice and policy and for future research (Levac et al., 2010).

## Results

**Figure 1.**
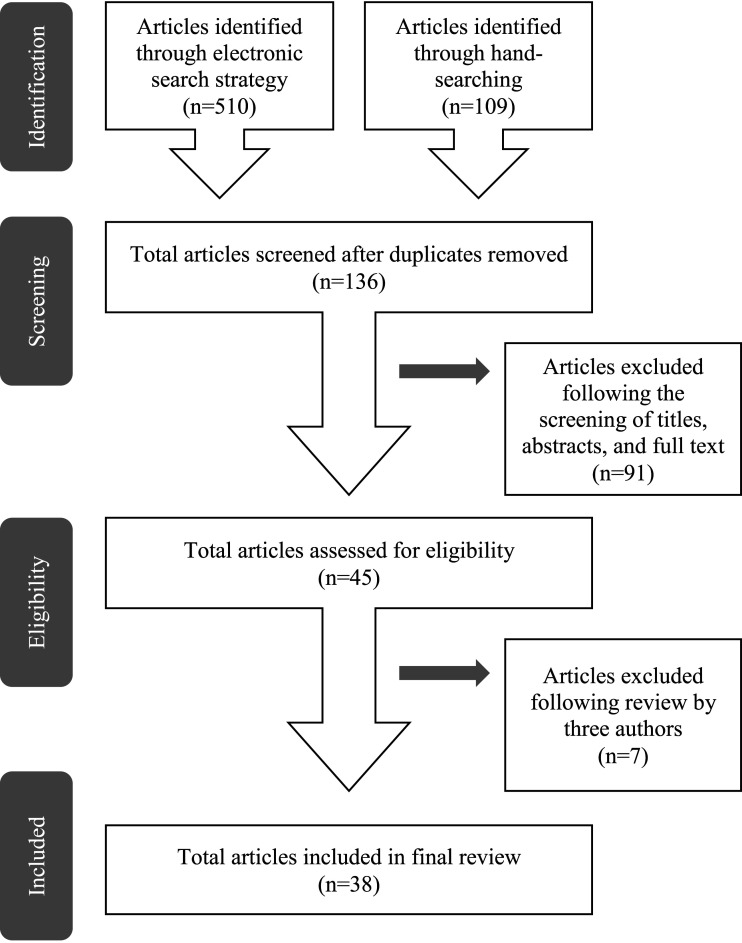
Flowchart of Screening Process.

The electronic search resulted in 510 citations for review (including duplicates). Hand-searching identified an additional 109 titles. After removing duplicates and those that obviously did not meet inclusion criteria based on title, a total of 136 titles and abstracts were reviewed. Ninety-one articles were excluded following the screening abstracts, and full text. A total of 45 articles were then reviewed individually by three authors to make final determination, and 38 articles were selected for inclusion in the review (see Figure 1 for flowchart of screening process).

Table 1 includes location information from all studies included in our review.

**Table 1.** Number of Studies by Location.

Multi-National Projects	6 studies	10 European Countries: Austria, Denmark, France, Germany, Hungary, Ireland, Italy, Spain, Sweden and the UK (1) 15 African countries: Kenya, Botswana, South Africa, Tanzania, Cote d’Ivoire, Ethiopia, Senegal, Zambia, Nigeria, Zimbabwe, Malawi, Cameroon, Burkina Faso, The Gambia (1) 10 Latin American Countries – Argentina, Brazil, Chile Colombia, Cuba, Guatemala, Mexico, Panama, Peru, Venezuela (1) Argentina and Latin America (1) United States and Switzerland (1) England and Wales (1)
Single Nation Projects	32 studies	Australia (1) Brazil (1) Canada (3) Egypt (1) Finland (1) India (1) Korea (1) Malaysia (1) New Zealand (1) Tanzania (1) The Netherlands (1) United Kingdom (3) United States (16)

Methods used in the selected articles included surveys (19); qualitative interviews studies (13); case studies (5) using a mixture of survey, interview, and documentary analysis; as well as one study that used a deliberative writing approach (1). Research participants from the selected papers were either REB Chairs, REB members (including scientists, physicians, lay/community/non-scientists members, etc.), researchers, administrators, institutional officials, or consultants. Studies ranged in size from 19 participants to over 2000. Authors collated information around the four questions of interest related to REB membership expertise and training.

### Question 1: How do REBs Ensure They Have Appropriate Scientific Expertise?

Of the 38 articles selected for the review, 21 addressed our first question regarding scientific expertise. Firstly, the articles indicated that scientific expertise on a board was thought to be very important (Bell et al., 1998; Bento et al., 2011; Halila, 2014). It was noted, however, that there was a lack of resources/support for adequate expertise and training of board members (Milford et al., 2006; Rivera & Ezcurra, 2001). Secondly, only a minority of articles indicated that REBs had an insufficient range of expertise on boards to do their job in general (Berry et al., 2019; Catania et al., 2008b; Klitzman, 2013; Paul, 2000). Nevertheless, problems regarding scientific expertise were raised within many articles when the discussion concerned domains with which boards might have less familiarity (social sciences versus clinical sciences) or nuanced areas with ethical (pediatric, HIV, or mental health research), or scientific complexity (cutting edge technologies, data science, neuroscience, etc.) (Catania et al., 2008a; Favaretto et al., 2020; Klitzman, 2013; Milford et al., 2006; Sellers et al., 2020; Stroustrup et al., 2008; Thabane et al., 2005; Vitak et al., 2017).

The articles noted that gaps in expertise could be resolved by using experts external to the REB. Where this did occur, it was found to be helpful (Sirotin et al., 2010); although little was known about how frequently this occurred (Bell et al., 1998; Catania et al., 2008b). Others have suggested more people from specialty research areas should volunteer to be on REBs (Racine et al., 2011); and that in general REBs should collaborate more with each other to overcome the limitations of individual REB's membership (Homedes & Ugalde, 2015; Kim et al., 2003; Racine et al., 2011).

Concerns were raised about the potential for scientific expertise, or the evaluation of scientific aspects, to dominate REB decision-making. Some articles mentioned that those members with scientific expertise could overpower discussions, and sometimes to the detriment of non-expert members input (McNeill et al., 1994; Sirotin et al., 2010). Others indicated that REBs may not have a clear understanding or agreement of the relationship between scientific and ethical evaluation in the review process, allowing the scientific review to dominate or be at odds with the ethical review (Homedes & Ugalde, 2015; Humphreys et al., 2014b; Vitak et al., 2017).

### Question 2: How do REBs Ensure They Have Appropriate Ethical, Legal, and Regulatory Expertise?

Of the 38 articles selected for the review, 14 included research findings relevant to our second question regarding ethical, legal, and regulatory expertise. In the articles found for this review, ethics expertise was discussed either in relation to its absence or the need for training. Regulatory knowledge was discussed only once and legal expertise was not discussed at all.

One article that discussed the inclusion of bioethicists found that they were viewed favorably and that bioethicist members not only helpfully contributed to the evaluation of protocols, but provided education to the REB while doing so (DeRenzo & Wichman, 1990). Others called for greater inclusion of a range of members including ethicists (Hall, 1991; Ikingura et al., 2007; See et al., 2019). However, many studies conveyed the lack of ethics expertise of their REB's members, sometimes as a self-identified lack of expertise among members (de Vries & Forsberg, 2002; Hall, 1991; Rivera & Ezurra, 2001;), or lack of knowledge of relevant regulation (Johnson et al., 2015). This was tied to lamenting a lack of adequate training (Matar & Silverman, 2013) or calling for more/specific training (Chenneville et al., 2016; DeRenzo & Wichman, 1990).

### Question 3: How do REBs Ensure They Have Appropriate Diversity of People and Perspectives?

Of the 38 articles selected for the review, 11 contained information relevant to our third question regarding the representation of diverse people and perspectives. Many of the participants in the studies of our selected articles were identified racially as white (Anderson, 2006; Catania et al., 2008a; de Vries & Forsberg, 2002; Hayes et al., 1995), and in some cases predominantly male (Kim et al. ,2003; Matar & Silverman, 2013); one study that reviewed a subsample of 85 committees in the US setting found that while IRB Chairs were predominantly white, membership was gender balanced and was somewhat better in terms of ethnicity/race (Catania et al., 2008a); and one study found that lay or community membership (Cargill, 2018) was more diverse. Lack of diversity across the chairs and membership was discussed as problematic due to the potential inability of the REB to adequately consider participants’ values, experiences, or situations (Anderson, 2006; Hayes et al., 1995; Paul, 2000).

The articles called for the increased inclusion of lay/community members on REBs (de Vries & Forsberg, 2002; See et al., 2019; Sirotin et al., 2010) because of the great value they bring to the review (Hung et al., 2013), but also indicated the difficulty in recruiting and retaining them (Matar & Silverman, 2013; Porter, 1986).

Interestingly a study comparing non-scientist REB members and community advisory board (CAB) members found that non-scientists saw themselves negatively as lacking expertise, while CAB members viewed themselves positively as experts in relation to their community and experiences (Cargill, 2018). This self-perception may have an effect not only on recruiting non-scientist/lay members to REBs, but also in how comfortable they feel in speaking up, or in how their views are considered in review. This was supported by work that discussed physicians’ and scientists’ views as dominating discussions (Humphrey et al., 2014a, 2014b; Matar & Silverman, 2013).

### Question 4: How do REBs Ensure That Research Participants’ Perspectives are Appropriately Represented?

Of the 38 articles selected for the review, 14 contained information relevant to our fourth question regarding the inclusion of research participant (or alternatively community stakeholder) perspectives on REBs. Articles included in this review describe the value of insight into participant position and perspective, and yet REBs lack this insight (Sirotin et al., 2010; van Luijn et al., 2007). Where there was knowledge of participants’ views or experiences, this came either through community/lay members or contact with third party organizations (Cox et al., 2020).

Although non-scientist, non-affiliated REB members are often included to provide the research participant perspective, these individuals tend to see their main role as providing a lay, nonscientific perspective rather than as advocates for vulnerable populations or specific communities; most focus on the consent form and rely heavily on scientific members’ opinions regarding the rest of the research protocols (Anderson, 2006; Humphreys et al., 2014b). This reliance may be the result of a power imbalance or hierarchy within the committee where scientific members’ dominate discussions (Anderson, 2006; Klitzman, 2015; Matar & Silverman, 2013; McNeill et al., 1994). A 1986 survey of non-scientist/non-affiliated members indicated that the responsibility to represent the perspectives of participants ranked fairly low as a priority (Porter, 1986). Non-scientist and non-affiliated members on REBs frequently see themselves as laypersons lacking expertise; the vague and unspecified role leaves them to define themselves and their contributions in relation to other members and in terms of what they “are not” (Cargill, 2018). In a United Kingdom study of REB members, not all participants could clearly say whether they fell in the “expert” or “lay” category, and there was a spectrum of uncertainty about their roles (Humphreys et al., 2014a).

## Discussion

A review of empirical research on REB membership and expertise identified a small and diverse body of literature with studies from around the world. The articles selected for this review focused on a variety of issues relevant to who is on REBs and their qualifications to do the important work of research ethics review. London (2021) has recently argued that the quality of review and the consideration of social value is improved when committees are diverse, not only in the identities of their members, but also in their expertise, where lived experience, community knowledge, and/or research participant experience are a form of expertise. This diversity is recommended in many regulations, and a recent study in the United States suggests that REB Chairs and members agree (Churchill et al. 2022); that same study found, as did this review, that diversity is still lacking.

***Scientific Expertise.*** Many existing studies focus on very narrow types of expertise, asking specific questions about areas with high scientific (neuroscience, data science, etc.) or ethical (pediatric research, mental health research, HIV trials, etc.) complexity. While the selected studies suggest that generally respondents feel their REBs have sufficient expertise in general, the conclusion across multiple studies was that for emerging areas or fields with increased complexity, greater training or use of expertise was needed (either through recruitment or external consultants). Serpico et al.'s (2022) recent work on the use of external experts summarizes a range of research on the topic. They suggest that large committee memberships may not improve decision-making if all members are not active in discussions and that the use of external experts, when specific expertise is lacking, may be more beneficial (Serpico et al., 2022).

However, the logistics around the use of experts has not been well-documented or explored as was also first noted by Bell et al. (1998) and later Hedgecoe in a study of REBs in the United Kingdom (2020). Others have raised that external scientific review whether proactive (i.e., prior to REB review) or reactive (based on a recognized lack of expertise during a review) can come with its own challenges of quality and trust (Hedgecoe, 2020; Tolich & Smith, 2015).

***Ethical, Legal, and Regulatory Expertise.*** Existing research on ethical expertise reveals gaps in the literature regarding how to define, measure, and improve such expertise (Braun et al., 2020; Taylor, 2007). The inclusion of members with legal and regulatory expertise has neither been specified by many regulations, nor explored by researchers. While work by Babb (2020) has shown a rise in compliance culture and professionals in HRPP programs, this has not necessarily led to greater regulatory knowledge of REB members, as was found here. Instead, this shift separates regulatory review from other considerations, with much of the regulatory work happening prior to review by REB support/HRPP staff (Babb, 2020). Others have observed and argued that very little ethical analysis happens in REB deliberations, arguing that much discussion centers on interpretation of regulation or participant-facing documentation, and aside from the risk-benefit analysis little else recognizable as ethical discussion occurs (Hedgecoe, 2020; Klitzman, 2015; Moore & Donnelly, 2015; Stark, 2012; Trace & Kolstoe, 2017; van Lent et al., 2014). it is worth asking, however, whether this might reflect REB composition–that is, why would a committee potentially dominated by scientific expertise and with little ethics training engage in explicit ethical analysis and reasoning? One study in our review investigated the usefulness of having bioethicists on REBs (DeRenzo & Wichman, 1990). It was thought to be beneficial, not only for their contribution to deliberation (picking up on things unnoticed, providing more insight, etc.) but because they also provided education (DeRenzo & Wichman, 1990). This was further supported by the work of Sirotin et al. (2010). While many of these studies call for greater training on the ethical aspects of review, and we agree, it is worth considering whether a bioethicist member should also be a requirement.

***Diversity of People and Perspectives.*** REB membership is overwhelmingly male, white, and educated, and often lacks the makeup and backgrounds of the research subjects they hope to protect (Churchill et al., 2022). Lack of diversity in REB membership means that the full range of stakeholder values are not adequately included in REB deliberations. This is especially problematic for assessments related to context- and value-dependent considerations of research, such as informed consent and acceptable risks.

Despite regulatory requirements in many countries for the inclusion of lay, community, non-scientist, and other members on REBs, there is a lack of clarity regarding what such members are meant to contribute to deliberations and why such roles are important in the first place (Solomon, 2016). There is evidence that these types of members are recruited -non-systematically (Nicholls et al., 2023), and it has been suggested that the ways in which members can be selected undermine their roles. For example, they may be closely connected to the universities whose REBs they sit on and/or have had significant scientific or clinical experience (i.e., retired doctors or scientists) (Anderson, 2006; Cho & Billings, 1997; Nicholls et al., 2023). There is also evidence to suggest that the perspectives of non-scientists may be different than those of scientists and other professionals involved in research oversight. For example, in a study of healthy individuals’ assessment of typical research risks, participants identified risk as higher than it would be categorized by federal regulations (Roberts & Kim, 2017) providing some evidence that non-scientists can provide valuable input that is different and not necessarily obvious to other reviewers.

To fulfill their mandates, REBs must ensure that efforts to include diverse lay, public perspectives are genuine, meaningful, and robust. Clearer institutional policies outlining the roles and expectations of these members on REBs are needed. Such organization-specific policies should take into consideration the research portfolio of that institution and how members’ areas of expertise correspond, so non-scientific members can be more effective in their roles.

***Research Participants’ Perspectives.*** While many studies have focused on non-scientist and non-affiliated or lay REB members, the extent to which these individuals have experience as research participants, as suggested by the CIOMS guidelines, is unknown. Inclusion of research participant perspectives may add value to ethical deliberation, but this was not adequately explored in the literature we found.

## Best Practices

REBs should regularly assess their membership and be proactive about identifying gaps in expertise of all kinds. It would also be beneficial to formalize information gathering (at a REB level and potentially at a national level in each jurisdiction) regarding membership demographics, experience, expertise and training, as well as use of external experts. REBs and their support staff should make greater efforts to include lay and non-scientists members and research participant members not only in committee membership but in the discussions related to reviews.

## Research Agenda

A key limitation of this scoping review is also a major finding, namely that there are not many empirical studies on REB membership or the effect of membership on decisions. There have been no studies regarding the inclusion of research participant perspectives, an area where further work is urgently needed. A recent report by the Government Office of Accountability identified the need for measures of quality and effectiveness of REB review (GAO-23–104721) (Government Accountability Office, 2023). Such measures would facilitate assessment of the impact of membership expertise and diversity on review decisions. Development of such measures should include additional stakeholders beyond REB members, such as research subjects, researchers, policy makers, research funders, etc.

## Educational Implications

We echo the calls of many of the authors of studies we reviewed to improve education and support for all REB members, as the research landscape and regulations are constantly evolving, and new empirical research explores the experiences and perspectives of diverse research participants and the communities from where they are drawn. REB Chairs and HRPP staff could benefit from greater educational support regarding the recruitment and retention of different members, as well as the use of outside expertise. Those responsible for training REB professionals and members, as well as bioethicists and scientists, would benefit from understanding the issues of REB membership and expertise and the implications for deliberation. Scientist and clinician REB members need education regarding the reasons for and potential contributions of lay and non-scientists members. Lay and non-scientist members need training – but more importantly support – to ensure that their input is sought and heard.
